# Bis[(2-chloro-4-fluoro­benz­yl)triphenyl­phospho­nium] bis­(1,2,5-thia­diazole-3,4-dithiol­ato)nickelate(II)

**DOI:** 10.1107/S1600536812003625

**Published:** 2012-02-04

**Authors:** Zhou-Hua Zeng, Shui-Bin Yang

**Affiliations:** a College of Chemical Engineering, Huanggang Normal University, Huangzhou 438000, People’s Republic of China

## Abstract

The title ion-pair complex, (C_25_H_20_ClFP)_2_[Ni(C_2_N_2_S_3_)_2_], was obtained by the direct reaction of (4-F,2-ClBzTPP)^+^·Br^−^ [4-F,2-ClBzTPP^+^ is (2-chloro-4-fluoro­benz­yl)triphenyl­phos­pho­nium], NiCl_2_·6H_2_O and Na_2_tdas (tdas^2−^ is 1,2,5-thia­diazole-3,4-dithiol­ate) in methanol. The asymmetric unit of the title structure comprises one (4-F,2-ClBzTPP)^+^ cation and half of an [Ni(tdas)_2_]^2−^ complex anion, with the Ni^II^ ion situated on a center of symmetry, leading to a slightly distorted square-planar coordination of the latter. In the cation, the tetra­hedral angles around the P atom are nearly undistorted. In the crystal, the cations and anions are linked by C—H⋯S, C—H⋯N and C—H⋯Cl hydrogen bonds.

## Related literature

For background to complexes containing the [Ni(maleo­nitrile­dithiol­ate)_2_]^2−^ anion, see: Chen *et al.* (2010 ▶); Hou *et al.* (2008 ▶); Ni *et al.* (2005 ▶); Ren *et al.* (2002 ▶); Robertson & Cronin (2002 ▶); Xie *et al.* (2002 ▶); Zhou *et al.* (2011 ▶). For details of other square-planar Ni(1,2,5-thia­diazole-3,4-dithiol­ate)_2_ complexes, see: Awaga *et al.* (1994 ▶); Yamochi *et al.* (2001 ▶); Okuno *et al.* (2003 ▶); Ni *et al.* (2004 ▶); Zuo *et al.* (2009 ▶).
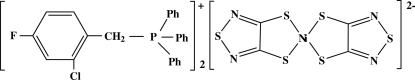


## Experimental

### 

#### Crystal data

(C_25_H_20_ClFP)_2_[Ni(C_2_N_2_S_3_)_2_]
*M* *_r_* = 1166.81
Triclinic, 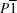

*a* = 9.4447 (11) Å
*b* = 12.1385 (15) Å
*c* = 13.1309 (16) Å
α = 71.447 (1)°
β = 83.601 (2)°
γ = 68.691 (2)°
*V* = 1329.6 (3) Å^3^
*Z* = 1
Mo *K*α radiation
μ = 0.81 mm^−1^
*T* = 291 K
0.19 × 0.15 × 0.11 mm

#### Data collection

Bruker SMART CCD diffractometer
Absorption correction: multi-scan (*SADABS*; Bruker, 2004 ▶) *T*_min_ = 0.861, *T*_max_ = 0.916
9694 measured reflections
4652 independent reflections
3807 reflections with *I* > 2σ(*I*)
*R*_int_ = 0.033

#### Refinement

*R*[*F* ^2^ > 2σ(*F* ^2^)] = 0.042
*wR*(*F* ^2^) = 0.118
*S* = 1.00
4652 reflections
322 parameters
H-atom parameters constrained
Δρ_max_ = 0.54 e Å^−3^
Δρ_min_ = −0.39 e Å^−3^

### 

Data collection: *SMART* (Bruker, 2004 ▶); cell refinement: *SAINT* (Bruker, 2004 ▶); data reduction: *SAINT*; program(s) used to solve structure: *SHELXTL* (Sheldrick, 2008 ▶); program(s) used to refine structure: *SHELXTL*; molecular graphics: *SHELXTL*; software used to prepare material for publication: *SHELXTL*.

## Supplementary Material

Crystal structure: contains datablock(s) I, global. DOI: 10.1107/S1600536812003625/wm2587sup1.cif

Structure factors: contains datablock(s) I. DOI: 10.1107/S1600536812003625/wm2587Isup2.hkl

Additional supplementary materials:  crystallographic information; 3D view; checkCIF report

## Figures and Tables

**Table:** 

Ni1—S1	2.1842 (9)
Ni1—S2	2.1966 (9)

**Table:** 

S1^i^—Ni1—S2	86.82 (3)
S1—Ni1—S2	93.18 (3)

Symmetry code: (i) 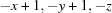.

**Table 2:** Hydrogen-bond geometry (Å, °)

*D*—H⋯*A*	*D*—H	H⋯*A*	*D*⋯*A*	*D*—H⋯*A*
C9—H9*A*⋯S1^ii^	0.97	2.71	3.635 (4)	160
C9—H9*B*⋯Cl1	0.97	2.66	3.129 (4)	110
C24—H24⋯N2^iii^	0.93	2.61	3.401 (7)	143

Symmetry codes: (ii) 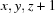; (iii) 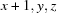.

**Fig. 1.**
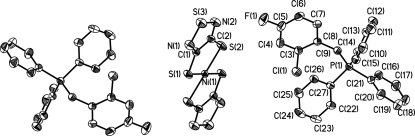
The molecular structure of (I), with atom labels and atoms displayed with displacement ellipsoids at the 30% probability level for all non-H atoms. The non-labelled atoms are generated by the inversion symmetry operation: -x + 1, -y + 1, -z.

**Fig. 2.**
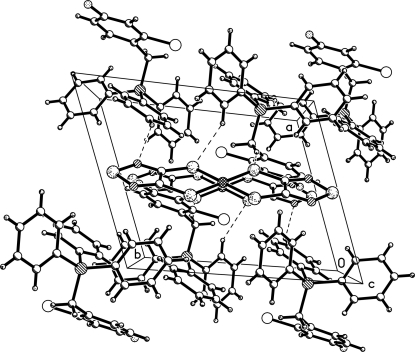
The crystal packing of (I), viewed approximately down the a axis, showing the network of molecules connected by non-classical hydrogen bonds (dashed lines).

## supplementary crystallographic information

### Comment

Transition metal complexes of bis(1,2-ditholene) and derivatives thereof have been extensively studied due to their potential applications in molecular materials showing superconducting, magnetic or optical properties (Robertson & Cronin, 2002; Ni *et al.*, 2005; Ren *et al.*, 2002). In recent years, much attention has been paid to the study of ion-pair complexes containing the [Ni(mnt)_2_]^n-^ (mnt is maleonitriledithiolate, n is 1 or 2) anion that possesses spin bistability with potential application as a molecular switch, in data storage or in displays (Chen *et al.*, 2010; Hou *et al.*, 2008; Xie *et al.*, 2002; Zhou *et al.*, 2011), while there is only few information available on complexes containing the [*M*(tdas)_2_]^n-^ (tdas is 1,2,5-thiadiazole-3,4-dithiolate, n is 1 or 2; *M* is a transition metal) anion (Awaga *et al.*, 1994; Yamochi *et al.*, 2001; Okuno *et al.*, 2003). Substantial efforts have been devoted for finding more suitable counter cations to tune the stacking in the crystal structures containing [*M*(tdas)_2_]^n-^ anions and also to obtain materials with interesting properties (Ni *et al.*, 2004; Zuo *et al.*, 2009). Substituted benzyl triphenylphosphonium as a cation has been proved to be suitable for this purpose. In this article we report on the preparation and crystal structure of the new ion-pair complex, [4-F,2-ClBzTPP]_2_[Ni(tdas)_2_] (I).

The molecular structure of (I) is shown in Fig. 1. There are one (2-chloro-4-fluorobenzyl)triphenylphosphonium and half of an [Ni(tdas)_2_]^2-^ anion in the asymmetric unit of (I). The nickel(II) ion of the complex [Ni(tdas)_2_]^2-^ anion is situated on a center of symmetry within a slightly distorted square-planar coordination. The Ni1—S1 and Ni1—S2 bond lengths are 2.1842 (9) Å and 2.1966 (9) Å, and the S1—Ni1—S2 bond angle within the five-membered metalla ring is 93.18 (3)°, similar to those observed for other structures with an [Ni(tdas)_2_]^2-^ anion (Okuno *et al.*, 2003; Zuo *et al.*, 2009). In the cation, the deviations of the F and Cl atoms from the C3—C8 benzene ring are 0.082 (2)Å and -0.029 (2) Å, respectively.

C—H···S, C—H···N and C—H···Cl hydrogen bonds between the anion and cation consolidate the crystal packing (Fig. 2, Table 2).

### Experimental

The title ion-pair complex was prepared by the direct reaction of 1:2:2 mol equiv. of NiCl_2_^.^6H_2_O, Na_2_tdas and (2-chloro-4-fluorobenzyl)triphenylphosphonium bromide in methanol. A brown product was obtained and purified through recrystallization from a mixed solution of methanol and water (yield: 86%). Brown block-sheped single crystals suitable for X-ray analysis were obtained by slow evaporation of the solvent at room temperature within 3 weeks.

### Refinement

All H-atoms were positioned geometrically and refined using a riding model with d(C—H) = 0.93 Å, *U*_iso_=1.2*U*_eq_ (C) for aromatic and 0.97 Å, *U*_iso_ = 1.2*U*_eq_ (C) for CH_2_ atoms.

### Crystal data

**Table:**

(C_25_H_20_ClFP)_2_[Ni(C_2_N_2_S_3_)_2_]	*Z* = 1
*M**_r_* = 1166.81	*F*(000) = 598
Triclinic, *P*1	*D*_x_ = 1.457 Mg m^−^^3^
Hall symbol: -P 1	Mo *K*α radiation, λ = 0.71073 Å
*a* = 9.4447 (11) Å	Cell parameters from 3884 reflections
*b* = 12.1385 (15) Å	θ = 2.3–26.0°
*c* = 13.1309 (16) Å	µ = 0.81 mm^−^^1^
α = 71.447 (1)°	*T* = 291 K
β = 83.601 (2)°	Block, brown
γ = 68.691 (2)°	0.19 × 0.15 × 0.11 mm
*V* = 1329.6 (3) Å^3^	

### Data collection

**Table:**

Bruker SMART CCD diffractometer	4652 independent reflections
Radiation source: fine-focus sealed tube	3807 reflections with *I* > 2σ(*I*)
Graphite monochromator	*R*_int_ = 0.033
φ and ω scans	θ_max_ = 25.0°, θ_min_ = 1.6°
Absorption correction: multi-scan (*SADABS*; Bruker, 2004)	*h* = −11→10
*T*_min_ = 0.861, *T*_max_ = 0.916	*k* = −14→14
9694 measured reflections	*l* = −15→15

### Refinement

**Table:**

Refinement on *F*^2^	Primary atom site location: structure-invariant direct methods
Least-squares matrix: full	Secondary atom site location: difference Fourier map
*R*[*F*^2^ > 2σ(*F*^2^)] = 0.042	Hydrogen site location: inferred from neighbouring sites
*wR*(*F*^2^) = 0.118	H-atom parameters constrained
*S* = 1.00	*w* = 1/[σ^2^(*F*_o_^2^) + (0.0368*P*)^2^ + 1.988*P*] where *P* = (*F*_o_^2^ + 2*F*_c_^2^)/3
4652 reflections	(Δ/σ)_max_ < 0.001
322 parameters	Δρ_max_ = 0.54 e Å^−^^3^
0 restraints	Δρ_min_ = −0.39 e Å^−^^3^

### Special details

**Table:**

Geometry. All e.s.d.'s (except the e.s.d. in the dihedral angle between two l.s. planes) are estimated using the full covariance matrix. The cell e.s.d.'s are taken into account individually in the estimation of e.s.d.'s in distances, angles and torsion angles; correlations between e.s.d.'s in cell parameters are only used when they are defined by crystal symmetry. An approximate (isotropic) treatment of cell e.s.d.'s is used for estimating e.s.d.'s involving l.s. planes.
Refinement. Refinement of *F*^2^ against ALL reflections. The weighted *R*-factor *wR* and goodness of fit *S* are based on *F*^2^, conventional *R*-factors *R* are based on *F*, with *F* set to zero for negative *F*^2^. The threshold expression of *F*^2^ > σ(*F*^2^) is used only for calculating *R*-factors(gt) *etc*. and is not relevant to the choice of reflections for refinement. *R*-factors based on *F*^2^ are statistically about twice as large as those based on *F*, and *R*- factors based on ALL data will be even larger.

### Fractional atomic coordinates and isotropic or equivalent isotropic displacement parameters (Å^2^)

**Table:**

	*x*	*y*	*z*	*U*_iso_*/*U*_eq_	
Ni1	0.5000	0.5000	0.0000	0.03811 (17)	
S1	0.58693 (12)	0.34739 (9)	−0.07028 (7)	0.0552 (3)	
S2	0.42586 (13)	0.38755 (9)	0.14777 (8)	0.0618 (3)	
S3	0.49400 (15)	0.04337 (10)	0.12386 (9)	0.0728 (3)	
Cl1	0.70371 (13)	0.46088 (10)	0.42942 (8)	0.0685 (3)	
F1	0.5385 (4)	0.1177 (3)	0.4145 (3)	0.1061 (10)	
N1	0.5634 (4)	0.1203 (3)	0.0147 (3)	0.0644 (9)	
N2	0.4341 (4)	0.1508 (3)	0.1854 (3)	0.0654 (9)	
P1	0.95727 (10)	0.22890 (8)	0.69433 (7)	0.0408 (2)	
C1	0.5392 (4)	0.2291 (3)	0.0242 (3)	0.0459 (8)	
C2	0.4655 (4)	0.2480 (3)	0.1213 (3)	0.0475 (8)	
C3	0.6640 (4)	0.3250 (3)	0.4940 (3)	0.0505 (8)	
C4	0.6126 (5)	0.2753 (4)	0.4295 (3)	0.0632 (11)	
H4	0.5992	0.3136	0.3559	0.076*	
C5	0.5826 (5)	0.1690 (4)	0.4779 (4)	0.0684 (11)	
C6	0.5948 (5)	0.1121 (4)	0.5864 (4)	0.0680 (11)	
H6	0.5686	0.0418	0.6172	0.082*	
C7	0.6469 (4)	0.1618 (4)	0.6486 (3)	0.0544 (9)	
H7	0.6565	0.1236	0.7224	0.065*	
C8	0.6859 (4)	0.2682 (3)	0.6042 (3)	0.0436 (8)	
C9	0.7547 (4)	0.3131 (3)	0.6737 (3)	0.0456 (8)	
H9A	0.7042	0.3038	0.7429	0.055*	
H9B	0.7376	0.4006	0.6404	0.055*	
C10	0.8961 (5)	0.0449 (4)	0.8608 (3)	0.0588 (10)	
H10	0.8138	0.1087	0.8753	0.071*	
C11	0.9250 (6)	−0.0750 (4)	0.9244 (3)	0.0738 (12)	
H11	0.8615	−0.0925	0.9819	0.089*	
C12	1.0475 (6)	−0.1698 (5)	0.9035 (4)	0.0797 (14)	
H12	1.0651	−0.2511	0.9461	0.096*	
C13	1.1436 (5)	−0.1448 (4)	0.8202 (4)	0.0717 (12)	
H13	1.2272	−0.2089	0.8075	0.086*	
C14	1.1167 (4)	−0.0246 (3)	0.7551 (3)	0.0553 (9)	
H14	1.1824	−0.0076	0.6990	0.066*	
C15	0.9911 (4)	0.0703 (3)	0.7741 (3)	0.0454 (8)	
C16	0.9476 (5)	0.3957 (4)	0.8019 (3)	0.0583 (10)	
H16	0.8426	0.4258	0.7933	0.070*	
C17	1.0149 (6)	0.4456 (5)	0.8554 (4)	0.0757 (13)	
H17	0.9553	0.5092	0.8832	0.091*	
C18	1.1704 (6)	0.4007 (5)	0.8674 (4)	0.0777 (13)	
H18	1.2152	0.4347	0.9032	0.093*	
C19	1.2600 (5)	0.3067 (5)	0.8274 (4)	0.0760 (13)	
H19	1.3650	0.2777	0.8352	0.091*	
C20	1.1938 (5)	0.2558 (4)	0.7757 (3)	0.0673 (11)	
H20	1.2540	0.1904	0.7501	0.081*	
C21	1.0368 (4)	0.3015 (3)	0.7614 (3)	0.0475 (8)	
C22	1.1227 (4)	0.3129 (3)	0.5183 (3)	0.0559 (9)	
H22	1.1355	0.3625	0.5553	0.067*	
C23	1.1813 (5)	0.3194 (4)	0.4163 (4)	0.0721 (12)	
H23	1.2353	0.3724	0.3855	0.087*	
C24	1.1614 (5)	0.2499 (5)	0.3607 (4)	0.0773 (14)	
H24	1.2011	0.2559	0.2919	0.093*	
C25	1.0820 (5)	0.1692 (4)	0.4052 (3)	0.0663 (11)	
H25	1.0678	0.1221	0.3662	0.080*	
C26	1.0242 (4)	0.1596 (3)	0.5083 (3)	0.0524 (9)	
H26	0.9721	0.1052	0.5394	0.063*	
C27	1.0450 (4)	0.2327 (3)	0.5651 (3)	0.0439 (8)	

### Atomic displacement parameters (Å^2^)

**Table:**

	*U*^11^	*U*^22^	*U*^33^	*U*^12^	*U*^13^	*U*^23^
Ni1	0.0364 (3)	0.0470 (4)	0.0314 (3)	−0.0127 (3)	0.0052 (2)	−0.0163 (2)
S1	0.0713 (6)	0.0579 (6)	0.0441 (5)	−0.0277 (5)	0.0236 (4)	−0.0269 (4)
S2	0.0861 (7)	0.0503 (5)	0.0480 (5)	−0.0237 (5)	0.0288 (5)	−0.0230 (4)
S3	0.0978 (9)	0.0573 (6)	0.0710 (7)	−0.0355 (6)	0.0188 (6)	−0.0257 (5)
Cl1	0.0798 (7)	0.0599 (6)	0.0587 (6)	−0.0315 (5)	−0.0064 (5)	0.0020 (5)
F1	0.123 (3)	0.127 (3)	0.108 (2)	−0.064 (2)	−0.0197 (19)	−0.056 (2)
N1	0.085 (2)	0.055 (2)	0.061 (2)	−0.0268 (18)	0.0149 (17)	−0.0296 (16)
N2	0.082 (2)	0.057 (2)	0.059 (2)	−0.0284 (18)	0.0207 (17)	−0.0200 (16)
P1	0.0422 (5)	0.0435 (5)	0.0385 (4)	−0.0172 (4)	0.0047 (4)	−0.0137 (4)
C1	0.0465 (19)	0.048 (2)	0.0452 (19)	−0.0146 (16)	0.0030 (15)	−0.0204 (16)
C2	0.0447 (19)	0.054 (2)	0.0430 (18)	−0.0168 (16)	0.0083 (15)	−0.0157 (16)
C3	0.046 (2)	0.054 (2)	0.050 (2)	−0.0155 (17)	0.0012 (16)	−0.0157 (17)
C4	0.058 (2)	0.079 (3)	0.052 (2)	−0.019 (2)	−0.0064 (18)	−0.022 (2)
C5	0.063 (3)	0.081 (3)	0.077 (3)	−0.029 (2)	−0.010 (2)	−0.036 (3)
C6	0.066 (3)	0.067 (3)	0.083 (3)	−0.037 (2)	−0.003 (2)	−0.021 (2)
C7	0.050 (2)	0.061 (2)	0.054 (2)	−0.0263 (18)	0.0026 (17)	−0.0123 (18)
C8	0.0367 (17)	0.0486 (19)	0.0444 (18)	−0.0121 (15)	0.0041 (14)	−0.0168 (15)
C9	0.0443 (19)	0.050 (2)	0.0436 (18)	−0.0154 (16)	0.0045 (15)	−0.0182 (16)
C10	0.059 (2)	0.069 (3)	0.044 (2)	−0.024 (2)	0.0023 (17)	−0.0088 (18)
C11	0.083 (3)	0.078 (3)	0.052 (2)	−0.040 (3)	−0.002 (2)	0.006 (2)
C12	0.098 (4)	0.063 (3)	0.068 (3)	−0.037 (3)	−0.025 (3)	0.011 (2)
C13	0.075 (3)	0.056 (3)	0.073 (3)	−0.013 (2)	−0.019 (2)	−0.010 (2)
C14	0.053 (2)	0.054 (2)	0.056 (2)	−0.0165 (18)	−0.0012 (17)	−0.0143 (18)
C15	0.0471 (19)	0.052 (2)	0.0387 (17)	−0.0216 (16)	−0.0003 (15)	−0.0098 (15)
C16	0.059 (2)	0.064 (2)	0.060 (2)	−0.022 (2)	0.0012 (18)	−0.029 (2)
C17	0.083 (3)	0.083 (3)	0.077 (3)	−0.032 (3)	−0.001 (2)	−0.041 (3)
C18	0.094 (4)	0.091 (3)	0.068 (3)	−0.050 (3)	−0.013 (3)	−0.025 (3)
C19	0.066 (3)	0.088 (3)	0.081 (3)	−0.029 (3)	−0.018 (2)	−0.027 (3)
C20	0.054 (2)	0.074 (3)	0.074 (3)	−0.015 (2)	−0.012 (2)	−0.027 (2)
C21	0.052 (2)	0.052 (2)	0.0408 (18)	−0.0230 (17)	−0.0022 (15)	−0.0108 (15)
C22	0.060 (2)	0.051 (2)	0.058 (2)	−0.0253 (19)	0.0099 (18)	−0.0135 (18)
C23	0.072 (3)	0.073 (3)	0.068 (3)	−0.038 (2)	0.025 (2)	−0.011 (2)
C24	0.075 (3)	0.085 (3)	0.055 (2)	−0.020 (3)	0.029 (2)	−0.016 (2)
C25	0.071 (3)	0.072 (3)	0.053 (2)	−0.016 (2)	0.013 (2)	−0.030 (2)
C26	0.054 (2)	0.057 (2)	0.048 (2)	−0.0209 (18)	0.0103 (16)	−0.0185 (17)
C27	0.0429 (18)	0.0435 (19)	0.0437 (18)	−0.0155 (15)	0.0081 (14)	−0.0132 (15)

### Geometric parameters (Å, º)

**Table:**

Ni1—S1^i^	2.1842 (9)	C10—H10	0.9300
Ni1—S1	2.1842 (9)	C11—C12	1.379 (7)
Ni1—S2^i^	2.1966 (9)	C11—H11	0.9300
Ni1—S2	2.1966 (9)	C12—C13	1.371 (7)
S1—C1	1.736 (4)	C12—H12	0.9300
S2—C2	1.737 (4)	C13—C14	1.381 (6)
S3—N2	1.644 (3)	C13—H13	0.9300
S3—N1	1.655 (3)	C14—C15	1.386 (5)
Cl1—C3	1.752 (4)	C14—H14	0.9300
F1—C5	1.361 (5)	C16—C21	1.373 (5)
N1—C1	1.300 (4)	C16—C17	1.384 (5)
N2—C2	1.324 (5)	C16—H16	0.9300
P1—C27	1.798 (3)	C17—C18	1.376 (7)
P1—C21	1.799 (3)	C17—H17	0.9300
P1—C15	1.802 (4)	C18—C19	1.370 (7)
P1—C9	1.816 (3)	C18—H18	0.9300
C1—C2	1.427 (5)	C19—C20	1.368 (6)
C3—C8	1.393 (5)	C19—H19	0.9300
C3—C4	1.394 (5)	C20—C21	1.394 (5)
C4—C5	1.361 (6)	C20—H20	0.9300
C4—H4	0.9300	C22—C27	1.379 (5)
C5—C6	1.368 (6)	C22—C23	1.380 (6)
C6—C7	1.375 (5)	C22—H22	0.9300
C6—H6	0.9300	C23—C24	1.349 (6)
C7—C8	1.401 (5)	C23—H23	0.9300
C7—H7	0.9300	C24—C25	1.391 (6)
C8—C9	1.503 (5)	C24—H24	0.9300
C9—H9A	0.9700	C25—C26	1.388 (5)
C9—H9B	0.9700	C25—H25	0.9300
C10—C11	1.371 (6)	C26—C27	1.400 (5)
C10—C15	1.395 (5)	C26—H26	0.9300
			
S1^i^—Ni1—S1	180.00 (4)	C10—C11—H11	119.8
S1^i^—Ni1—S2^i^	93.18 (3)	C12—C11—H11	119.8
S1—Ni1—S2^i^	86.82 (3)	C13—C12—C11	120.3 (4)
S1^i^—Ni1—S2	86.82 (3)	C13—C12—H12	119.9
S1—Ni1—S2	93.18 (3)	C11—C12—H12	119.9
S2^i^—Ni1—S2	180.00 (8)	C12—C13—C14	120.3 (4)
C1—S1—Ni1	103.26 (11)	C12—C13—H13	119.8
C2—S2—Ni1	102.65 (12)	C14—C13—H13	119.8
N2—S3—N1	98.37 (16)	C13—C14—C15	119.4 (4)
C1—N1—S3	106.9 (3)	C13—C14—H14	120.3
C2—N2—S3	106.6 (3)	C15—C14—H14	120.3
C27—P1—C21	109.46 (16)	C14—C15—C10	120.0 (3)
C27—P1—C15	109.54 (16)	C14—C15—P1	120.6 (3)
C21—P1—C15	109.54 (16)	C10—C15—P1	119.2 (3)
C27—P1—C9	108.25 (16)	C21—C16—C17	119.6 (4)
C21—P1—C9	109.81 (16)	C21—C16—H16	120.2
C15—P1—C9	110.22 (16)	C17—C16—H16	120.2
N1—C1—C2	114.5 (3)	C18—C17—C16	119.8 (4)
N1—C1—S1	125.5 (3)	C18—C17—H17	120.1
C2—C1—S1	120.1 (3)	C16—C17—H17	120.1
N2—C2—C1	113.6 (3)	C19—C18—C17	120.9 (4)
N2—C2—S2	125.7 (3)	C19—C18—H18	119.6
C1—C2—S2	120.7 (3)	C17—C18—H18	119.6
C8—C3—C4	121.8 (4)	C20—C19—C18	119.5 (4)
C8—C3—Cl1	121.3 (3)	C20—C19—H19	120.2
C4—C3—Cl1	116.8 (3)	C18—C19—H19	120.2
C5—C4—C3	117.8 (4)	C19—C20—C21	120.3 (4)
C5—C4—H4	121.1	C19—C20—H20	119.8
C3—C4—H4	121.1	C21—C20—H20	119.8
F1—C5—C4	117.8 (4)	C16—C21—C20	119.8 (4)
F1—C5—C6	119.0 (4)	C16—C21—P1	122.2 (3)
C4—C5—C6	123.2 (4)	C20—C21—P1	117.9 (3)
C5—C6—C7	118.1 (4)	C27—C22—C23	119.8 (4)
C5—C6—H6	120.9	C27—C22—H22	120.1
C7—C6—H6	120.9	C23—C22—H22	120.1
C6—C7—C8	122.0 (4)	C24—C23—C22	120.9 (4)
C6—C7—H7	119.0	C24—C23—H23	119.6
C8—C7—H7	119.0	C22—C23—H23	119.6
C3—C8—C7	116.9 (3)	C23—C24—C25	120.7 (4)
C3—C8—C9	122.8 (3)	C23—C24—H24	119.7
C7—C8—C9	120.2 (3)	C25—C24—H24	119.7
C8—C9—P1	112.3 (2)	C26—C25—C24	119.4 (4)
C8—C9—H9A	109.2	C26—C25—H25	120.3
P1—C9—H9A	109.2	C24—C25—H25	120.3
C8—C9—H9B	109.2	C25—C26—C27	119.4 (4)
P1—C9—H9B	109.2	C25—C26—H26	120.3
H9A—C9—H9B	107.9	C27—C26—H26	120.3
C11—C10—C15	119.5 (4)	C22—C27—C26	119.8 (3)
C11—C10—H10	120.3	C22—C27—P1	120.8 (3)
C15—C10—H10	120.3	C26—C27—P1	119.2 (3)
C10—C11—C12	120.4 (4)		

Symmetry code: (i) −*x*+1, −*y*+1, −*z*.

### Hydrogen-bond geometry (Å, º)

**Table:**

*D*—H···*A*	*D*—H	H···*A*	*D*···*A*	*D*—H···*A*
C9—H9*A*···S1^ii^	0.97	2.71	3.635 (4)	160
C9—H9*B*···Cl1	0.97	2.66	3.129 (4)	110
C24—H24···N2^iii^	0.93	2.61	3.401 (7)	143

Symmetry codes: (ii) *x*, *y*, *z*+1; (iii) *x*+1, *y*, *z*.
